# Personalized diet order compliance is associated with an improved functional independence measure (FIM) score in elderly patients: An eight-week follow-up study in a convalescent hospital

**DOI:** 10.1371/journal.pone.0314394

**Published:** 2024-12-03

**Authors:** Jung Min Cho, Song Woo Ha, Minji Son

**Affiliations:** 1 K-Food Industry Research Institute, Jeonju University, Jeonju-si, Republic of Korea; 2 Department of Medicine, Central Convalescent Hospital, Daegu, Republic of Korea; 3 Department of Food and Nutrition, Central Convalescent Hospital, Daegu, Republic of Korea; Jashore University of Science and Technology (JUST), BANGLADESH

## Abstract

It is important to establish the relationship between appropriate nutritional intake and improvements in activities of daily living (ADLs) in elderly hospitalized patients. This prospective observational study aimed to investigate diet order compliance (DOC) during 8 weeks of hospitalization and calculate the odds of improved functional independence measure (FIM) scores for high- and low-DOC groups using covariate-adjusted models in geriatric convalescent hospitals. The study subjects were elderly inpatients (>65 years old) with degenerative disease who consumed prescribed oral meals (Functional Oral Intake Scale (FOIS) = 6/7) and who did not receive physical/occupational therapy. The personalized diet order was prescript, and the DOC was calculated using dietitian-monitored daily intake data. The 73 patients were divided into a low-DOC group (< 84.0%, n = 35) and a high-DOC group (≥ 84.0%, n = 38) on the basis of the median DOC (84.0%, average for 8 weeks). Twenty (52.6%) high-DOC patients and nine (25.7%) low-DOC patients experienced motor-FIM improvements (P = 0.017). After 8 weeks, the change in motor-FIM in the high-DOC group (1.6±0.3) was greater than that in the low-DOC group (0.3±0.1; P = 0.001). According to the baseline and nutrition-intake-adjusted model of multiple logistic regression analysis, in the high-DOC group, the motor-FIM improvement OR was 5.102 (95% CI: 1.100–16.233, P = 0.036), and the total-FIM improvement OR was 5.273 (95% CI: 1.102–25.238, P = 0.037). High compliance with individualized nutritional prescriptions can increase FIM scores in clinical settings. Thus, comprehensive approaches to increase dietary compliance are needed for elderly long-term care patients.

## Introduction

Frail elderly inpatients are susceptible to malnutrition due to reduced intake, which may lead to deterioration in mobility and activities of daily living (ADLs) [1]. Furthermore, undernutrition in elderly individuals is associated with negative health outcomes, resulting in a decline in functional status [2], quality of life [3], and mortality [4]. Additionally, once impairments and a decline in functional status have occurred, complete recovery of mobility function may be difficult for elderly people with degenerative diseases [5, 6].

In practical settings, we often encounter patients in long-term institutionalization who cannot receive active rehabilitation treatment, such as physical and occupational therapy units, for socioeconomic reasons, and many elderly patients undergo nutritional interventions as essential medical care. For patients hospitalized for convalescent treatment, nutritional intake often depends entirely on the meal prescriptions provided at the hospital; thus, health professionals have made efforts to provide appropriate nutrition to long-term hospitalized patients to prevent malnutrition [7, 8]. However, even when effective systems such as nutritional screening, nutritional diet prescription, meal preparation and delivery are established to provide adequate nutrition in clinical settings [9], discrepancies have been observed between prescribed nutrition and actual nutritional intake, with patients consuming less than the prescribed amount [10, 11]. Thus, it is essential to examine and monitor the associations between nutritional status and compliance with individualized diet prescriptions in hospitalized patients to determine whether their nutritional intake is adequate during long-term hospitalization. However, there is a lack of research on the health benefits associated with high personalized diet order compliance (DOC) during long-term hospitalization of elderly individuals who are prone to impaired nutritional status [12, 13].

Moreover, in terms of quality of life management for these elderly patients [14], adequate nutrition was shown to have a positive effect on ADLs [15]. The influence of nutritional intake on the improvement of ADLs is reported as an improvement in the functional independence measure (FIM) score, which is a widely used functional performance measure of observational ADL scales in rehabilitation. The populations for whom the FIM score is most applicable include patients with stroke (for whom the FIM was originally designed [14]), dementia [16], parkinsonism [17], and malignant cancer [18]. Previous studies have focused mainly on the relationships of nutritional status (but not actual nutritional intake) with FIM score changes [19–21]. However, there is insufficient research on how DOC and actual intake (prescriptions vs. intake ratios) in hospitalized patients correlate with improvements in FIM scores in geriatric clinical settings.

The objectives of our study were to 1) investigate DOC during 8 weeks of hospitalization, 2) compare FIM scores and FIM score changes between individuals with high and low DOCs, and 3) use covariate-adjusted models (basic information and nutritional intake amounts) to calculate the odds of improved FIM scores for the high- and low-DOC groups of patients over 65 years of age who did not receive rehabilitation, were hospitalized for convalescent purposes, and were treated with oral meals.

## Methods and materials

### Study design and subject recruitment

This was a cross-sectional and observational study using real-world data, and the follow-up period was 8 weeks from admission. The observation period was designed on the basis of the average length of hospital stay of 59.8 days reported in a previous study [22], which was conducted with elderly patients with dementia and cerebrovascular, motor and disuse syndromes in a nursing hospital. To minimize differences in seasonal factors such as periodic cold, influenza, and seasonal food selection, this study was intensively conducted over a short study period of 6 months. Patients admitted to the hospital between June 2023 and October 2023 were eligible, and the follow-up of the last patient in our study was completed in December 2023. The patients in this study were admitted to a 200-bed convalescent hospital located in Daegu, Republic of Korea, and were provided nonacute-phase long-term care. When designing this study, considering the number of admissions/discharges at our hospital and the 6-month study period, we expected that approximately 80 patients would be included among our research subjects. Subjects who agreed to participate in the study and met the inclusion criteria below were enrolled by the attending physician.

### Subject inclusion criteria

**Fig 1:**
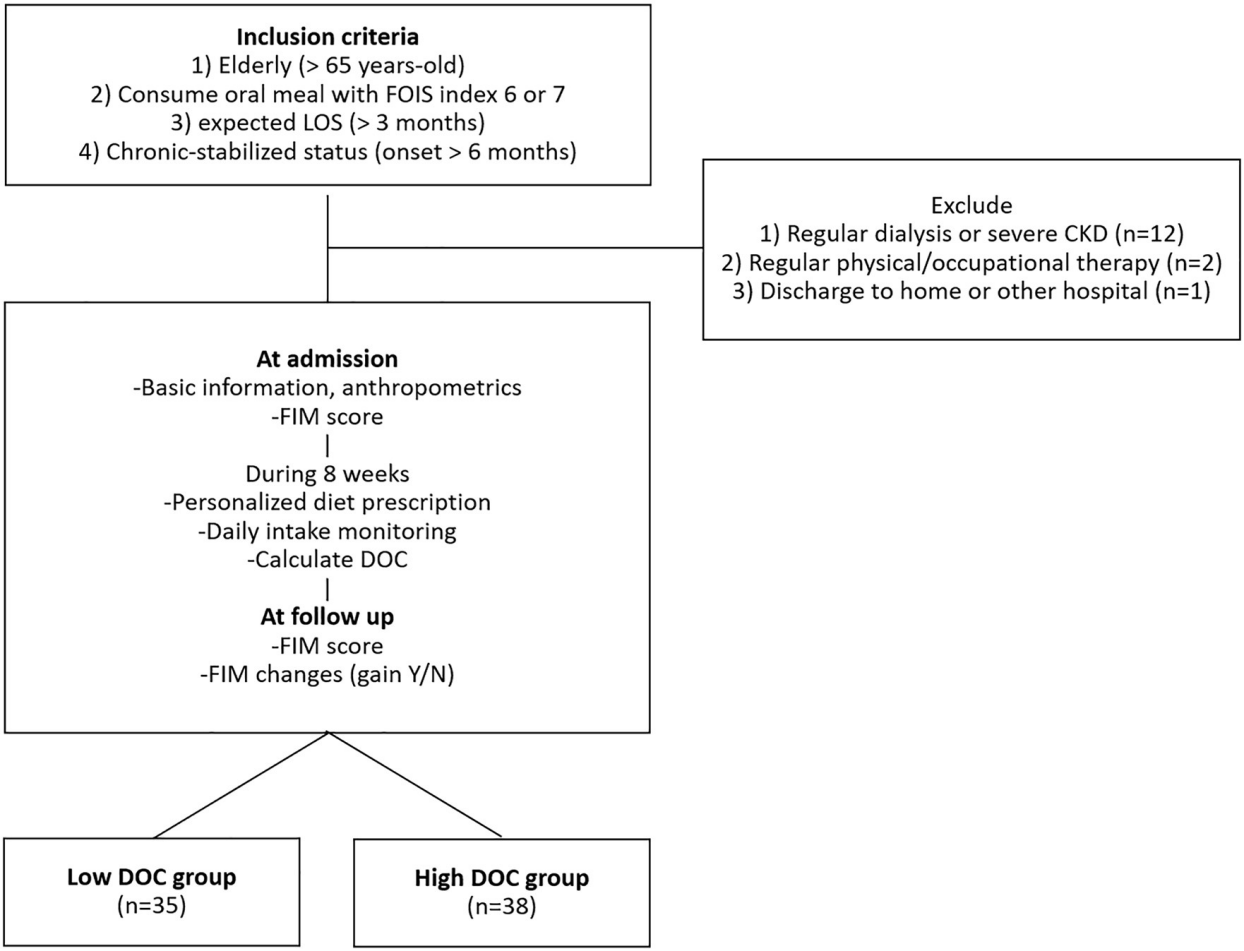
Flow diagram of our study participants. FOIS; Functional Oral Intake Scale, CKD; chronic kidney failure, FIM; functional independence measure, DOC; diet order compliance.

Our study subjects were stabilized elderly patients who were hospitalized for the purpose of recovery. The inclusion criteria were as follows: (i) older than 65 years of age admitted to our hospital for recovery; (ii) oral intake of 6 (total oral diet without special preparation but specific food limitations) or 7 (total oral diet with no restriction) according to the Functional Oral Intake Scale (FOIS, [23]); (iii) an expected length of stay (LOS) of more than three months; and (iv) chronic disease with an onset date greater than 6 months prior to the start of the study. The exclusion criteria were as follows: (i) aged < 65 years, (ii) receiving regular dialysis or diagnosed with chronic kidney failure, and (iv) receiving regular (more than 2 hours per week) professional physical, occupational or speech therapy. After the patients were selected, baseline data were collected at admission, and follow-up data were collected at 8 weeks. The flow diagram of the current study is shown in Fig 1.

### Determination of group size

The sample size was determined and calculated with reference to functional outcome score gain results from another clinical trial that showed statistically significant results when intensive nutritional supplementation was compared with the standard group [24]. Because our statistical goal was to identify a valid superior-FIM gain mean in the high DOC group, we performed the following step-by-step calculations using a two-sample *t* test power calculation. According to the superiority test based on that reference study, the change (improved) value in m-FIM was 16.71±9.64 (mean ± standard deviation) in the standard group, which was lower than that in the intensive group (24.25±11.83%). The difference in deltas between the two groups (effect size (*d*) of 7.54) was used. The results indicated that a minimum of 26 subjects per group was needed, and we estimated a dropout ratio of 30%. Thus, we selected 34 participants per group and aimed to recruit at least 68 participants to increase the statistical power of the test (basic assumptions included a significance level (α) of 0.05 using a two-sided test, type 2 error (β) set to 0.2, the power of the test maintained at 80%, and a 1:1 allocation ratio based on the median).

### Energy requirements and diet orders

In our study, after nutritional status, underlying diseases and consultation from a registered dietician (RD) were considered, the attending physician prescribed a personal diet according to the following three steps. The first priority was total energy. We determined the total caloric intake per kg of body weight (BW) according to two weight criteria. In a previous study including hospitalized elderly patients with an average age of 79.7 years [25], the suggested energy requirements for those with a body mass index (BMI) >21.0 kg/m^2^ and ≤21.0 kg/m^2^ were 18.4 kcal/kg/day and 21.4 kcal/kg/day, respectively, and the estimated energy requirements for men and women were similar. Second, protein was supplied at 1.0 g/kg/day to maintain a positive nitrogen balance. This is because, in a previous study targeting elderly hospitalized patients (aged 65–99 years), the protein intake range for maintaining a neutral or positive nitrogen balance was 0.99±0.24 g/kg to 1.06±0.28 g/kg per kg per day [26]. Third, the carbohydrate:protein:fat (C:P:F) ratio was adjusted based on the patient’s underlying disease. Specifically, excessive intake of carbohydrates was limited in diabetic patients (not consuming more than 55% of the recommended daily energy from carbohydrates, [27, 28]); salt intake was limited in hypertensive patients (in the case of salt restriction, it is necessary to provide no more than 2000 mg of sodium per day [29]); and high protein intake was prescribed if the nutritional support team determined that it was necessary (in the case of recent weight loss, severe malnutrition or underweight status, 1.0 g/kg protein was provided [30, 31]). In summary, the diets prescribed for patients included a diabetes mellitus (DM) diet, a low-salt (LS) diet, a diabetes mellitus and low-salt (DMLS) diet, and a high-protein (HP) diet. The details are presented in Table 1. The prepared Korean-style meal was delivered to the hospital room 3 times a day (breakfast at 07:00, lunch at 12:00, and dinner at 17:30).

**Table 1:** Diet type prescription indications.

Diet type	DM	LS	DMLS	HP
Indication/status	diabetes mellitus	hypertension, heart failure, liver cirrhosis with ascites	diabetic patients requiring sodium intake control	underweight, muscle weakness
Energy(/day)	18.4 kcal/kg (BMI greater than21.0 kg/m^2^) or 21.4 kcal/kg (BMI of 21.0 kg/m^2^ or less); and multiple physical activity			
C:P:F* ratio (%)	**~55**:15–20:20–25	65:15:20	~55:15–20:20–25	60:**20~**:20
Other	fiber 25–30 g/day, limit simple sugar	sodium <2000 mg/day	fiber 25–30 g/day, limit simple sugar, sodium <2000 mg/day	Protein 1.2–1.5 g/kg/day

*The approximate ratios and adjusted macronutrient ratios are in bold. C:P:F; carbohydrate: protein: fat, DM; diabetes mellitus diet, LS; low-salt diet, DMLS; diabetes mellitus and low-salt diet, HP; high-protein diet, BMI; body mass index. The above caloric and nutrient contents were divided into three meals, and a prepared Korean-style meal was delivered to the hospital room (breakfast at 07:00, lunch at 12:00, and dinner at 17:30).

### Basic information and measurements

Patients’ height and weight were assessed at admission as part of a routine check-up by a nurse. If height measurement was not possible, the most recent measurement or self-reported height was used. Using height (cm) and weight (kg) data, BMI was calculated (equation; [32]). Considering the main causes of disability, patients were divided into four causative disease groups: stroke (cerebral infarction, cerebral hemorrhage, subarachnoid hemorrhage, and stroke of unknown cause), dementia, Parkinson’s disease, and other disease (disuse syndrome, traumatic accident (fall, traffic accident), fracture, and general weakness). Because many of our patients were bedridden, the risk of pressure ulcers was also assessed by registered ward nurses using the Branden scale [33]. The Branden scale for predicting pressure ulcers is presented as follows: very high risk (total score of 9 or less), high risk (total score of 10–12), moderate risk (total score of 13–14), mild risk (total score of 15–18), and no risk (total score of 19–23).

### Cognitive decline and physical disability assessment

The Clinical Dementia Rating (CDR) is a scale that measures the overall cognitive, social, and self-care function of elderly patients [34]. The scale has been widely adapted for clinical research worldwide. In Korea, Choi et al. developed a Korean version of the CDR [35]. Additionally, it is highly evaluated as a tool for not only rating dementia severity but also diagnosing mild cognitive impairment, and the tool has been demonstrated to assess the severity of cognitive dysfunction more accurately than the Mini-Mental State Examination [36], which is relatively more affected by literacy or educational experience [37]. For dementia patients, we assessed only six domains of the CDR (memory, orientation, judgment and problem solving, community affairs, home and hobbies, and personal care) through patient and caregiver interviews. Each domain is rated on 5 levels of impairment: 0 (none), 0.5 (questionable), 1 (mild), 2 (moderate), and 3 (severe). However, an exception is that personal care is rated on a 4-point scale ranging from 0 to 3. In total, our subjects were assessed for cognitive ability, with CDR values ​​of 0, 0.5, 1, 2, and 3, and the rating scores were recorded in the admission electronic medical records by the nurse. The CDR scores for dementia patients are shown in S1 Table.

### Nutritional status assessment

All patients underwent nutritional screening upon admission via the Patient-Generated Subjective Global Assessment (PG-SGA) tool [38]. The numerical PG-SGA score is a clinical tool used to assess a patient’s nutritional status on the basis of history and physical examination findings. A previous study reported that the qualities of the diet and life of cerebral infarction patients are related to nutritional status, which was evaluated by the PG-SGA [39]. In another study, nutritional status at admission was a significant explanatory variable for FIM at discharge [40]; thus, we considered the study subjects’ baseline nutritional status as a covariate. The patients and patient family members were allowed to complete the subjective questionnaire parts of the PG-SGA, and the professional component of the PG-SGA was completed by a registered dietitian. After scoring the tool, nutritional status was categorized into three groups: stage A, well nourished; stage B, moderate or suspected malnutrition; and stage C, severely malnourished.

### Anorexia and appetite assessment

Elderly patients often suffer from psychogenic anorexia, which may contribute to poor dietary compliance [41]. Considering the need for appetite evaluation in individuals, the Simplified Nutritional Appetite Questionnaire (SNAQ) was administered during the admission evaluation through interviews with patients and dietitians. The SNAQ is a self-assessment nutritional screening tool that predicts weight loss and could be used to screen older people at risk of malnutrition or malnourishment [42]. It comprises four question items (appetite, feeling of satiety, taste, and the number of meals per day) answered on a 5-point scale. The sum of each item in the SNAQ ranges from 4 to 20 points, with lower scores indicating poor appetite [43], and previous studies employed a SNAQ Simplified score of ≤ 14 out of 20 as defining anorexia/appetite loss [44].

### Nutritional intake monitoring and DOC

During the study period of 8 weeks, the RD visually estimated the daily intake amount. Visual estimation by professionals is commonly used in clinical settings to evaluate meal intake through the estimation of plate waste [45]. Each meal was inspected on delivery and on completion, and consumption was estimated (on an 11-point scale from 0 to 10 [46]) for each component (e.g., rice, soup, noodles, meat and fish, eggs, tofu, vegetables and fruits, sauce, dairy products) of every meal. This dietary intake monitoring was converted to energy and nutrient intake using CAN-Pro 3.0, a commonly used nutrient analysis software program in Korea [47], based on the recipe for each menu. If intake data were missing for each meal, the data were imputed as the average of all the subjects for each breakfast, lunch, or dinner of that day. In our study, daily DOC generally refers to the intake-to-prescription ratio. The detailed derivation of the $DOC(\%)=\left(\frac{E\;intake\left(kcal\right)}{E\;supply\left(kcal\right)}+\frac{C\;intake\left(g\right)}{C\;supply\left(g\right)}+\frac{P\;intake(g)}{P\;supply\left(g\right)}+\frac{F\;intake\left(g\right)}{F\;supply\left(g\right)}\right)/4$. Each subject’s final DOC value was analyzed by calculating the 8-week average daily DOC. After the intake and prescribed amounts of the four dietary components (energy, carbohydrate, protein, and fat) were compared, the DOC (%) was calculated. Because this study had an 8-week observation period, each subject’s final DOC value was analyzed by calculating the 8-week average daily DOC. As the median 8-week average DOC of all study subjects was 84.0%, subjects with a low average DOC (under 84.0%, under the median value of the 8-week average DOC of all study subjects) were assigned to the low-DOC group (n = 35), and subjects with a high average DOC (over 84.0%, over the median value of the 8-week average DOC of all study subjects) were assigned to the high-DOC group (n = 38).

### Assessment with the functional independence measure (FIM)

The FIM, a universally standardized ADL evaluation method [14], was performed at admission and at follow-up to measure changes in ADLs and FIM scores. The FIM is a generic tool for measuring physical impairment and disability grade not only in patients with stroke but also in patients with dementia, parkinsonism, and other atrophic diseases [48]. The FIM is divided into two components, the motor-FIM (m-FIM) and the cognitive-FIM (c-FIM), and the sum of the two components is added to derive the total-FIM (t-FIM) [49, 50]. In particular, t-FIM and m-FIM improvements have been used as outcome indicators for convalescent patients with stroke who are older than 60 years of age [51]; furthermore, these measures have also been shown to be useful indicators of both motor and cognitive disabilities in patients with Alzheimer’s disease (AD) and vascular dementia [16]. Thus, the FIM score was evaluated at admission and at 8 weeks as a tool to evaluate the ADLs of our patients hospitalized by a general practitioner. FIM improvements were defined as positive change scores (delta (Δ)) when the FIM score at admission was subtracted from that at 8 weeks. FIM scores are rated by doctors or physical therapists on a 7-point ordinal scale ranging from 1 (complete dependence) to 7 (independence). The FIM score comprises 2 sections: m-FIM (13 items)—self-care (eating, grooming, bathing, dressing (upper), dressing (lower), toileting), sphincter control (bladder and bowel), transfers (bed/chair/wheelchair, toilet, tub/shower), and locomotion (walk/wheelchair and stairs); c-FIM (5 items)—communication (comprehension and expression) and social cognition (social interaction, problem solving, and memory). The total maximum score (t-FIM) is 126, and the minimum score is 18 [49].

### Ethical review

This study was approved by the Institutional Review Board of Jeonju University (jjIRB-230503-HR-2022-1212) and was conducted in accordance with the tenets of the Declaration of Helsinki. Written informed consent for data collection was obtained from all the subjects at admission, and all the collected data were deidentified for research purposes. Written informed consent, including the protection of medical information, was obtained from the attending physician. The study was conducted in accordance with the regulations of the Medical Service Act and Personal Information Protection Act of Korea.

### Statistical analyses

The basic statistical design of this study was to 1) compare the change in m-FIM and t-FIM scores between the low- and high-DOC groups for eight weeks and 2) determine the association (odds ratio) between FIM improvements and the DOC. Variables and data are presented as numerical values and percentages (categorial variables) and means ± standard errors (SEs, noncategorical variables). The *P* values of basic characteristics, nutritional intake, and FIM score changes according to the DOC group were derived from Pearson’s chi-square test (categorical variables) or ANOVA tests (continuous variables). Additionally, in ANOVA, when covariate adjustment was necessary, the baseline data of each variable were used as covariates. Bivariate correlation analyses were conducted, and Pearson’s product‒moment coefficient of correlation was used to determine the correlations among changes in the FIM score, DOC, and nutritional intake. Multiple logistic regression analysis was used to determine the odds ratios (ORs) and 95% confidence intervals (CIs) for FIM improvements (positive FIM score change for 8 weeks) according to the DOC in both the crude model and the adjusted model. Sex, age, diagnosis, ulcer risk grade, nutritional status (PG-SGA), energy, carbohydrate, protein, and fat intake were considered covariates. All the data were analyzed via SPSS version 26.0 (IBM/SPSS Corp., Chicago, IL, USA), and a two-tailed P value less than 0.05 was considered to indicate statistical significance.

## Results

### Basic characteristics of the subjects

Table 2 outlines the basic characteristics of all the included patients. The 73 patients were divided into the low-DOC group (< 84.0%, n = 35) and the high-DOC group (≥ 84.0%, n = 38) on the basis of the median DOC (84.0%, average for 8 weeks). The mean ages of the two groups were 82.26±0.99 and 81.37±1.22 years, and the mean BMIs were 20.88±0.69 kg/m^2^ and 20.24±0.62 kg/m^2^, respectively. Sex, causative disease, diet order type, nutritional status, pressure ulcer risk, and SNAQ score distributions did not significantly differ between the two groups.

**Table 2:** Basic characteristics of the study participants.

	Low DOC (n = 35)	High DOC (n = 38)	*P*
Age, mean (year-old)	82.26±0.99	81.37±1.22	0.580
Sex, n (%)			0.442
Male	8 (22.9)	12 (31.6)	
Female	27 (77.1)	26 (68.4)	
BMI, mean (kg/m^2^)	20.88±0.69	20.24±0.62	0.488
Causative disease*, n (%)			0.185
Post stroke	7 (20.0)	8 (21.1)	
Dementia	23 (65.7)	17 (44.7)	
Parkinson’s disease	2 (5.7)	3 (7.9)	
Other	3 (8.6)	10 (26.3)	
Diet order type, n (%)			0.401
DM	10 (28.6)	8 (21.1)	
LS	10 (28.6)	9 (23.7)	
DMLS	5 (14.3)	3 (7.9)	
HP	10 (28.6)	18 (47.4)	
Nutritional assessment (PG-SGA), n (%)			0.293
Well-nourished	5 (14.3)	10 (26.3)	
Moderate malnourished	18 (51.4)	20 (52.6)	
Severely malnourished	12 (34.3)	8 (21.1)	
Pressure ulcer risk (Branden), n (%)			
Mean score	16.63±0.45	17.42±0.57	0.281
Very high/High risk	2 (5.7)	3 (7.9)	0.103
Very high/High risk	3 (8.6)	7 (18.4)	
Mild/No risk	20 (85.7)	28 (73.6)	
Anorexia assessment (SNAQ), mean	11.85±0.64	10.44±0.53	0.751

Mean±standard error (SE) or the number of subjects. *P* values were derived from Pearson tests (categorical variables) and ANOVA tests (continuous variables). *P* < 0.05 was considered significant.

*Stroke (cerebral infarction, cerebral hemorrhage, subarachnoid hemorrhage, and stroke of unknown cause), dementia, Parkinson’s disease, and other disease (disuse syndrome, traumatic accident (fall, traffic accident), fracture, general weakness). PG-SGA, Patient-Generated Subjective Global Assessment, is categorized into three groups: stage A, well nourished; stage B, moderate or suspected malnutrition; and stage C, severely malnourished. The brand scale for predicting pressure ulcers is as follows: very high risk (total score of 9 or less), high risk (total score of 10–12), moderate risk (total score of 13–14), mild risk (total score of 15–18), and no risk (total score 19–23). SNAQ, simplified nutritional appetite questionnaire.

### DOC and daily energy and nutrient intake

Table 3 shows the 8-week comparison of the ratios of DOC and daily energy and nutrient intake between the low- and high-DOC groups. The minimum DOC was 40.0%, and the maximum DOC was 100.0% in all patients. The mean DOC of the high-DOC group was 89.9±0.9%, which was approximately 15% greater than that of the low-DOC group. The average daily energy intake of the high-DOC group was 1474.0±44.4/day, which was significantly greater than that of the low-DOC group (1195.5±58.2 kcal/day, P = 0.001). Additionally, the daily intake of carbohydrates, protein, and fat in the high-DOC group was greater than that in the low-DOC group (P = 0.001).

**Table 3:** Diet order compliance and daily energy and nutrient intake during 8 weeks of hospitalization.

	Low DOC (n = 35)	High DOC (n = 38)	*P*
Diet order compliance (DOC)			
median (min–max range, %)	84.0 (40.0–100.0)		
mean (%)	74.1±1.8	89.9±0.9	0.001
Nutritional intake (/day)			
Energy (kcal)	1195.5±58.2	1474.0±44.4	0.001
Carbohydrates (g)	175.3±9.6	220.4±5.5	0.001
Protein (g)	52.2±3.5	68.2±2.5	0.001
Fat (g)	26.6±1.7	34.3±1.2	0.001

Mean±standard error (SE) or the number of subjects. *P* values were derived from ANOVA tests (continuous variables) between two groups. *P* < 0.05 was considered significant.

### FIM improvement frequency and FIM score change according to the DOC

Table 4 shows the comparisons of FIM improvement frequency and FIM score changes according to the DOC. In the high-DOC group, twenty (52.6%) patients experienced m-FIM improvement, whereas nine (25.7%) patients in the low-DOC group experienced m-FIM improvement (P = 0.017). The change in the m-FIM of the high-DOC group after 8 weeks was 1.6±0.3, which was significantly greater than that of the low-DOC group (0.3±0.1; P = 0.001). Additionally, the t-FIM change at 8 weeks in the high-DOC group was 2.0±0.4, which was significantly greater than the 0.4±0.1 in the low-DOC group (P = 0.001). Because the FIM score at admission can influence FIM improvements [52], the changes in the FIM scores at 8 weeks were compared between the two groups using baseline data as a covariate. A baseline-adjusted between-group comparison at 8 weeks revealed significant differences in m-FIM individual components—selfcare, sphincter control, transfers, and locomotion—and c-FIM individual components—communication and social cognition factors (P< 0.05). In addition, the baseline-corrected m-FIM and t-FIM at week 8 were 41.6 ± 2.0 and 58.5 ± 2.5, respectively, in the high-DOC group and were significantly greater than the 35.3 ± 1.3 and 51.4 ± 1.6, respectively, in the low-DOC group (P = 0.001).

**Table 4:** Comparisons of FIM gain frequency and FIM score changes according to diet order compliance.

Low DOC (n = 35)			High DOC (n = 38)		*P* ^*a*^	*P* ^*b*^	*P* ^*c*^
0 week		8 week	0 week	8 week			
m-FIM gain, n (/%)	9/25.7		20/52.6		0.017		
t-FIM gain, n (/%)	15/42.9		24/63.1		0.066		
m-FIM subtotal	35.4±1.4	35.3±1.3	40.0±1.9	41.6±2.0	0.084	0.002	
*change*	0.3±0.1		1.6±0.3				0.001
Selfcare	18.1±0.7	18.0±0.7	18.8±0.9	19.7±0.9	0.208	0.001	
Sphincter control	5.5±0.3	5.6±0.3	6.9±0.5	6.9±0.4	0.005	0.586	
Transfers	8.1±0.4	8.0±0.4	9.4±0.5	9.8±0.6	0.098	0.001	
Locomotion	3.7±0.2	3.7±0.2	4.8±0.3	5.2±0.3	0.028	0.002	
c-FIM sub total	16.1±0.6	16.1±0.6	16.5±0.7	16.9±0.7	0.258	0.007	
*change*	0.0±0.1		0.4±0.1				0.007
Communication	6.9±0.2	6.9±0.2	7.2±0.3	7.3±0.3	0.211	0.031	
Social cognition	9.2±0.4	9.3±0.4	9.3±0.5	9.6±0.4	0.884	0.042	
t-FIM	51.4±1.7	51.4±1.6	56.5±2.4	58.5±2.5	0.075	0.001	
*change*	0.4±0.1		2.0±0.4				0.001

Mean±standard error (SE) or the number of subjects. *P*^*a*^ values were derived from Pearson tests (categorical variables) and ANOVA tests (continuous variables) between the two groups. *P*^*b*^ values were derived from baseline adjusted ANOVA at follow-up between the two groups. The *P*^*c*^ values were derived by baseline adjusted ANCOVA for differences between the two groups. *P* < 0.05 was considered significant. m-FIM; motor FIM score; t-FIM; total FIM score.

### Correlation between changes in FIM score and DOC

Bivariate correlation analysis revealed that the DOC was positively related to the m-FIM change (r = 0.416, P<0.001) and t-FIM change (r = 0.399, P<0.001). However, no significant correlation with the amount of energy (calories) or macronutrient intake was observed.

### Associations between FIM improvements and the DOC

The OR analysis of the FIM score according to the DOC is presented in Table 5. The above results revealed that 1) the frequency of FIM improvements in the high-DOC group was greater than that in the low-DOC group and that 2) DOC was more strongly correlated with m-FIM and t-FIM improvements than with nutritional intake. Thus, OR analysis evaluated the relationship between high DOC and FIM improvements in the following three models: the crude model (no covariates, reference; the DOC group in each model = 1.0), Model 1 (sex, age, diagnosis, ulcer risk grade, and nutritional status were used as covariates), and Model 2 (Model 1 + energy, carbohydrate, protein, and fat intake). According to the crude model, the OR of m-FIM improvement was 5.905 (95% CI: 2.048–17.021) for the high-DOC group (P = 0.001). In Model 1, which was adjusted for the basic characteristics of the patients, the OR of m-FIM improvement was 3.148 (95% CI: 1.139–8.700) for the high-DOC group (P = 0.027). In Model 2, which was additionally adjusted for nutritional intake as a covariate, the OR of m-FIM improvements was 5.102 (95% CI: 1.100–16.233, P = 0.036), and that of t-FIM improvements was 5.273 (95% CI: 1.102–25.238) for the high-DOC group (P = 0.037).

**Table 5:** Odds ratios of FIM gain and diet order compliance.

		m-FIM gain			t-FIM gain	
	OR	CI	*P*	OR	CI	*P*
Crude	**5.905**	2.048–17.021	0.001	2.942	0.815–10.622	0.099
Model 1^†^	**3.148**	1.139–8.700	0.027	3.041	0.665–13.894	0.151
Model 2^††^	**5.102**	1.100–16.233	0.036	**5.273**	1.102–25.238	0.037

The values are odds ratios (ORs) and 95*%* confidence intervals (CIs). *P* values were derived from the multiple logistic regression analysis. The low-compliance group was used as a reference (Refer. = Low DOC = 1.00).

^†^Adjusted Model 1; sex, age, diagnosis, ulcer risk grade, and nutritional status (PG-SGA) were included as covariates.

^††^Model 2; model 1 variables + energy, carbohydrate, protein, and fat intake were included as covariates. *P* < 0.05 was considered significant, and significant ORs are shown in bold.

## Discussion

It is important to establish the relationship between appropriate nutritional intake and improvements in ADLs in elderly hospitalized patients. Most importantly, this study revealed that 1) high compliance with diet prescriptions in elderly people hospitalized for convalescent purposes was associated with improved FIM scores and that 2) this association became stronger even after adjusting for caloric and macronutrient intake. Our findings are worth reporting because 1) DOC was derived by collecting real-world data on RD-monitored personalized diet order prescriptions and daily intake amounts for each patient over 8 weeks, although previous studies simply analyzed the intake amount of a single day in the hospital [53]; and 2) this focused study included elderly chronic disease patients with oral intake during an intensive study period (6 months of an 8-week follow-up study), whereas other studies included patients with dysphagia/enteral tube feeding [54], patients with a wide time frame (median length of stay more than 150 days) [55], or patients with multiple diseases in the acute phase [56].

Rehabilitation treatment is also indispensable for improving FIM, but we prioritized more practical aspects in our research framework. This study investigated the relationship between FIM improvements and nutritional intake in patients not receiving rehabilitation treatment, whereas previous studies focused on analyzing the effects of physical and occupational therapy treatment along with nutritional intake [57]. Previous studies have emphasized the importance of rehabilitation treatment in comprehensive nursing care, and the effects of concurrent nutritional interventions have been difficult to evaluate independently. Despite improvements in accessibility to medical care in Korea, limited coverage and benefit levels, resulting in high out-of-pocket costs, are barriers to accessing comprehensive medical services [58, 59]. In practical situations, limiting the burden of hospital expenses and individual insurance coverage for long-term hospitalized elderly people can prevent them from receiving all ideal treatments, including active rehabilitation physical therapy. Therefore, the framework of this study aims to verify the effectiveness of functional improvement through dietary interventions (diet, dietary intervention, and physician dietary prescription), which are almost fully covered by Korea’s national public health insurance, which targets patients who do not receive separate rehabilitation therapy.

A novel part of our research is that we derived the ORs for t-FIM and m-FIM improvements according to the DOC after adjusting for explanatory variables. Although no previous studies have performed OR analyses of FIM-DOC, it has been reported that better nutritional status and nutritional supplementation can improve FIM. In a previous randomized controlled trial [24], intensive nutritional supplementation (240 calories, 11 g of protein vs. standard; 127 calories, 5 g of protein) improved motor functions (FIM and 2- and 6-minute walk tests) in 102 undernourished patients. Their subjects included acute rehabilitation patients who coincided with the start of rehabilitation therapies (physical, occupational, speech) while on a nutritional supplementation routine, but the considered caloric count was a rough subjective estimation of nutritional intake. In a rehabilitation center study with convalescent patients with cerebrovascular disorders with poor nutritional status, patients who achieved improved nutritional status (assessed using the Geriatric Nutritional Risk Index (GNRI)) had improved t-FIM [20]. Although rehabilitation therapy was implemented in that study, greater FIM efficiency and improvements in the GNRI and increased energy intake were independently associated with the multivariate stepwise regression analysis. Additionally, in a multicenter retrospective cohort study of 702 stroke patients [21], undernutrition (determined by the controlling nutritional status score) was an independent predictor of poor m-FIM improvement.

Additionally, a nutritional support team (NST) combined with active rehabilitation treatment has shown that a comprehensive approach leads to better FIM improvement. According to Hidaka’s study on patients with a FOIS of 5 (a total oral diet with multiple consistencies), a comprehensive approach for reducing malnutrition in bed-bound patients with Alzheimer’s disease improved their ability to perform ADLs [15]. In addition to physical therapy (moved the patient in a wheelchair every day with full assistance, taking her out of her room to increase cognitive, motor, and sensory stimulation) and occupational therapy (adjusted the patient’s eating environment—the table’s height and position and eating posture—and trained her to use eating utensils, such as eating behavior rehabilitation), the RD on that research team prescribed a personalized diet and introduced oral supplements during the subsequent 3-week intervention; t-FIM increased from 18 to 30, of which m-FIM increased from 13 to 15 with increased oral intake function.

A key issue in the current study was whether a high DOC was more helpful than a low DOC. Although we were unable to identify clear evidence for this finding, we suggest that higher DOC was associated with improved FIM scores even after nutritional intake was treated as a covariate. First, the basic reason could be that our study subjects’ nutritional prescriptions (energy and nutrients) were different for each individual. In this study, we prescribed 22 kcal per kg of body weight, and the amount of prescribed energy was further reduced for obese patients. In elderly individuals, energy requirements decrease due to decreased body weight and activity [25, 60]; therefore, consuming less energy or nutrients does not mean that the subjects are undernourished or nutritionally deficient. In addition, although Sato’s study targeted acute patients within one week of stroke onset, protein intake and FIM score at discharge were not significantly associated in their regression analysis [46]. According to a report by a registered dietician, Shimazu [45], more frequent dietary adjustments and prescriptions to ensure adequate nutrition in hospitalized patients, rather than the sufficiency of specific nutrients, were independently associated with m-FIM scores at discharge (β = 0.104, P = 0.045). Furthermore, considering that our subjects were elderly patients with underlying diseases requiring diet adjustments (the four-diet type; DM, LS, DMLS, and HP), they may have been provided with a diet limited in certain nutrients. For example, the diabetic patients in our study consumed less than 55% of their daily energy. Even with a relatively low intake of carbohydrates, it is possible to obtain adequate nutrition if the intake rate of individually prescribed meals is high, which may have helped FIM improvement.

Second, oral nutrition may lead to independent physical and occupational training, potentially resulting in improved ADL function. In the present study, high compliance with meal prescriptions was related to the act of eating three meals a day at regular times, and consuming a large volume might be related to the many occupational/physical activities at mealtime. In a previous retrospective cohort study with 415 middle cerebral artery infarction patients aged 39–101 years [61], the eating-FIM score was the factor with the greatest predictive ability among the 18 single FIM items for home discharge (area under the receiver operating characteristic curve = 0.830, 95% CI = 0.787–0.874). Additionally, the OR of the eating FIM score was 1.280 (95% CI 1.102–1.488), and the cutoff score was 5 for the outcome destination, which indicates a better ADL outcome. Although we were not able to examine the volume of intake and time taken to consume meals, it is presumed that chewing, swallowing, holding and using utensils (cutlery, plates, cups, etc.), and preparing meals at the bedside may have contributed to FIM improvement. If in-depth studies can measure changes in muscle mass, bone structure, muscle mass, muscle strength, and body fat composition via bioelectrical impedance analysis or dual energy X-ray absorptiometry, clearer biological reasons for FIM gain with high DOC may be obtained.

Moreover, the average DOC over 8 weeks for the current study subjects reached a median of 84%. The low median DOC in our subjects might have been attributed to impaired nutritional conditions (INCs), possibly because of poor appetite [62] and decreased swallowing function [63] in elderly patients. A previous meta-analysis reported that the incidence of INC in poststroke patients was 21% (95% CI: 12–31) in the late subacute phase and 72% (95% CI: 41–100) in the chronic phase [64]. Low nutritional intake in hospitalized elderly individuals has often been reported in other studies. In a previous study conducted at a general hospital in Australia [53], only 41% of inpatients aged 65 years or older reported resting energy expenditure during hospitalization, and the mean energy intake of patients was 1220 kcal/day. In addition, in elderly patients, enteral feeding may be helpful as an adjuvant therapy. However, we limited oral intake to patients with a FOIS score of 5 or higher, which may have contributed to the low average DOC. Campaigns that reduce the gap between planned nutritional support and reality, such as managing nutrition wards or nutrition care units; enhancing nutrition education, training, and competencies; improving the dining environment; and providing dietary advice [10], could further increase patients’ actual intake in clinical settings without increasing rehabilitation treatment.

This study has other limitations. First, the short intensive research period in a single center and the lack of classification by disease group may have biased our outcomes. Caution is needed when attempting to generalize these findings because posthospitalization treatment policies might differ from those of other institutions. Additionally, the small sample size and large frequency of FIM improvements in this study can be expected to have a greater odds ratio [65]. Thus, statistical considerations are required when interpreting the sizes of ORs. Second, although we tried to include as many variables as possible as covariates, variables that were not included that can affect nutritional status and FIM outcomes could be potential biases (e.g., other underlying diseases, activity levels in the hospital, walking ability, understanding of the importance of nutritional intake, individual digestion ability/olfactory or taste disorders, personal use of dietary supplements, and other socioeconomic/education backgrounds). The OR value of our study was greater than 5, which may include the possibility of a positive correlation of nearly all factors during the convalescent period. Thus, more measurement data are needed. Third, although the DOC cannot be randomly assigned due to medical ethics issues, it would represent an opportunity to obtain better scientific evidence if DOC interventions and randomization could be implemented. In the future, a prospective all-case study that considers nutritional interventions, including enteral feeding and comprehensive rehabilitation therapy, should be performed.

The practical implications of this study are as follows. First, all professionals involved in the treatment of patients, especially dietitians, must make efforts to increase patients’ dietary compliance to help achieve FIM improvements in hospitals. Patients receiving nutrition support team (NST) management maintain the same level of ability to perform ADLs as do general patients despite their more severe condition [66], suggesting that comprehensive nutritional support is important. Second, from the patient’s perspective, they should try to achieve maximal compliance with the prescribed hospital diet, remembering that better compliance with dietary prescriptions is helpful for achieving improved FIM and preventing sarcopenia [15]. Third, we suggest that high compliance with hospital meals, even without the help of physical or occupational therapy, can lead to a reduction in economic medical costs. For example, greater ADL improvement was associated with greater home discharge in convalescent stage-malnourished elderly patients [55]. Instead of home discharge, consecutive transfers to other hospitals or longer hospital stays after admission not only result in greater medical expenses but also put patients at risk for infectious diseases or deterioration to a malignant state; therefore, improving ADLs by improving the DOC can contribute to reducing social costs.

## Conclusion

In conclusion, high compliance with individualized nutritional prescriptions in hospitals can increase FIM scores in frail elderly individuals. Therefore, comprehensive approaches to increase dietary compliance can help increase ADLs and quality of life in elderly long-term care patients.

## Supporting information

S1 Table Distribution of clinical dementia ratings among dementia patients. (DOCX)

S1 Checklist PLOSOne human subjects research checklist. (DOCX)

S1 File Institutional review board approval. (DOCX)
