# Supplementary material for: Personalized diet order compliance is associated with an improved functional independence measure (FIM) score in elderly patients: An eight-week follow-up study in a convalescent hospital
Source: PLoS One. 2024 Dec 3;19(12):e0314394. doi: 10.1371/journal.pone.0314394 (PMC11614235; doi:10.1371/journal.pone.0314394)
Supplement: S1 Table — (DOCX) [file pone.0314394.s001.docx]

Supplemental table 1. Clinical dementia rating distribution in dementia patients.

|  | Low DOC  (n=23) | High DOC  (n=17) | *P* |
| --- | --- | --- | --- |
| Clinical dementia rating (CDR^*^), n |  |  | NA |
| Questionable | 3 | 2 |  |
| Mild | 5 | 5 |  |
| Moderate | 8 | 6 |  |
| Severe | 7 | 4 |  |

CDR, Clinical dementia rating scale only in dementia patents. CDR 0=no dementia and CDR 0.5, 1, 2, or 3 indicates questionable, mild, moderate, or severe dementia respectively.
