# Supplementary material for: Personalized diet order compliance is associated with an improved functional independence measure (FIM) score in elderly patients: An eight-week follow-up study in a convalescent hospital
Source: PLoS One. 2024 Dec 3;19(12):e0314394. doi: 10.1371/journal.pone.0314394 (PMC11614235; doi:10.1371/journal.pone.0314394)
Supplement: S1 File — (DOCX) [file pone.0314394.s003.docx]

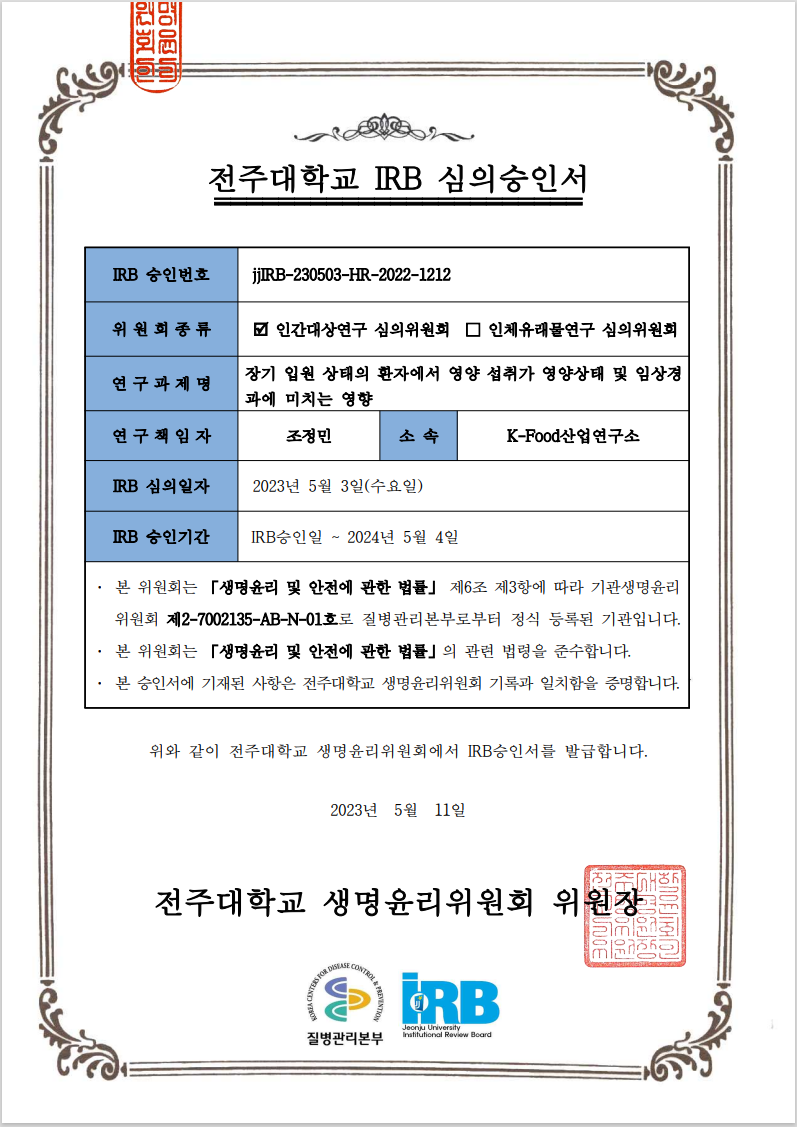


**PLOS Human Participants Research Checklist**

please upload (file type “Other”) the original approval document you received from your ethics committee. If the original document is in another language, please also provide an English translation.

**Jeonju University IRB Review Approval Certificate**

| IRB Approval No. | jjIRB-230503-HR-2022-1212 |
| --- | --- |
| Committee Types | Human Subjects Research Review Committee |
| Research Project Title | The Effect of Nutritional Intake on Nutritional Status and Clinical Outcome in Long-Term Hospitalized Patients |
| Research Director / Affiliation | Jung Min Cho / K-Food Industry Research Institute |
| IRB Approval Date | May 3, 2023 |
| IRB Approval Period | IRB Approval Date ~ May 4, 2024 |
| · This committee is an institution officially registered with the Korea Centers for Disease Control and Prevention as Institutional Bioethics Committee No. 2-7002135-AB-N-01 in accordance with Article 6, Paragraph 3 of the Bioethics and Safety Act.  · This committee complies with the relevant laws and regulations of the Bioethics and Safety Act.  · I hereby certify that the information contained in this approval is consistent with the records of the Jeonju University Bioethics Committee. | |

As above, the Jeonju University Bioethics Committee issues an IRB approval certificate.

May 11, 2023.

Chairman of the Jeonju University Bioethics Committee
